# Supplementary material for: A Bacteriophage Microgel Effectively Treats the Multidrug-Resistant Acinetobacter baumannii Bacterial Infections in Burn Wounds
Source: Pharmaceuticals (Basel). 2023 Jun 29;16(7):942. doi: 10.3390/ph16070942 (PMC10385199; doi:10.3390/ph16070942)
Supplement: Supplementary file 1 [file pharmaceuticals-16-00942-s001.zip › pharmaceuticals-2433565-supplementary.pdf]

Figure S1 Antibiotic sensitivity test of *Acinetobacter baumannii* (BHU/AB/39)

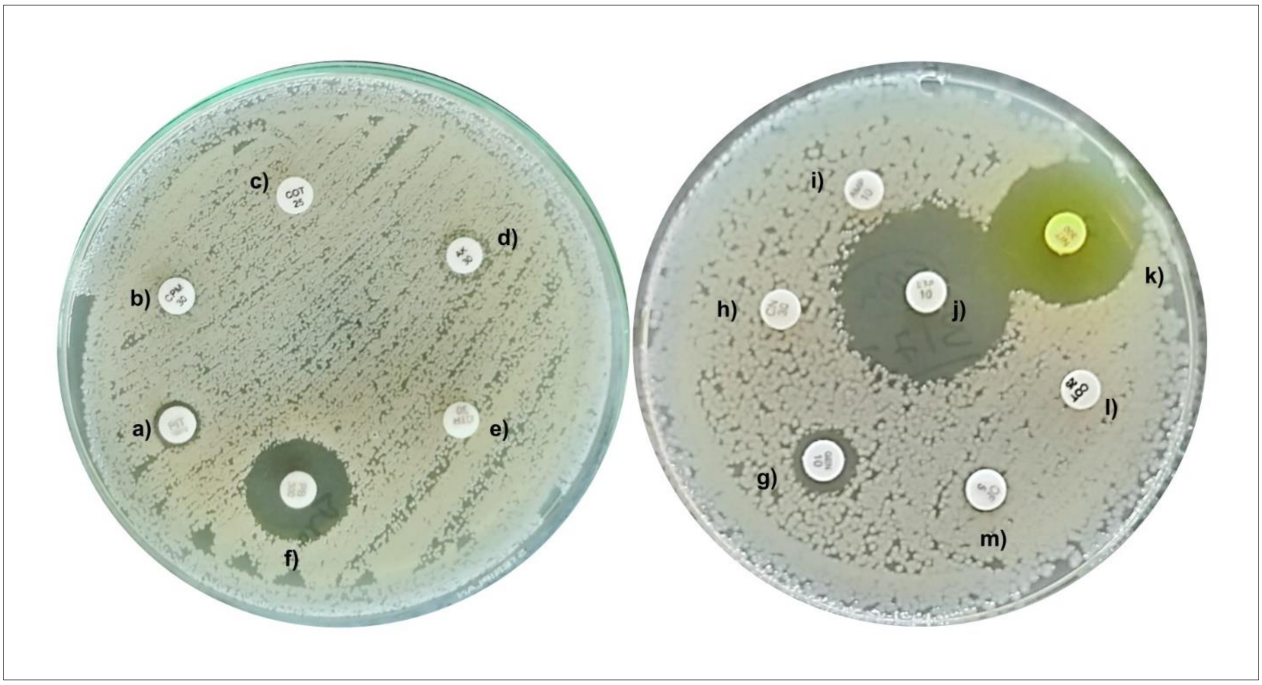

Table S1 Antibiotic sensitivity test of *Acinetobacter baumannii* (BHU/AB/39)

S. No	Symbol	Antibiotics	Standard Resistant Zone diameter (mm)	Result Zone diameter (mm) (n=3)	Result
a.	PIT	Piperacillin/ Tazobactam (100/10 mcg)	≤ 17	8±0.00	Resistant
b.	CPM	Cefepime (30mcg)	≤ 14	6±0.00	Resistant
c.	COT	Co–Trimoxazole (25mcg)	≤ 19	6±0.20	Resistant
d.	AK	Amikacin (30mcg)	≤ 14	9±0.00	Resistant
e.	CTR	Ceftriaxone (30mcg)	≤ 13	6±0.20	Resistant
f.	PB	Polymyxin–B (300 units)	≤ 19	16±0.00	Sensitive
g.	GEN	Gentamicin (10mcg)	≤ 12	10±0.00	Resistant
h.	CN	Cefalexin (30 mcg)	≤ 14	6±0.00	Resistant
i.	AMP	Ampicillin (10mcg)	≤ 13	6±0.00	Resistant
j.	ETP	Ertapenem (10mcg)	≤ 18	20±0.00	Sensitive
k.	NIT	Nitrofurantoin (300mcg)	≤ 15	21±0.01	Sensitive
l.	COT	Co–Trimoxazole (25mcg)	≤ 11	6±0.00	Resistant
m.	OF	Ofloxacin (5mcg)	≤ 22	7±0.00	Resistant

Table S2 Host range determination

S. No.	Bacterial strain	BPABΦ1
1	BHU/AB/17	-
2	BHU/AB/18	-
3	BHU/AB/19	-
4	BHU/AB/23	-
5	BHU/AB/24	-
6	BHU/AB/25	-
7	BHU/AB/26	-
8	BHU/AB/28	-
9	BHU/AB/30	-
10	BHU/AB/32	-
11	BHU/AB/36	-
12	BHU/AB/39	+
13	BHU/AB/40	-
14	BHU/AB/41	+
15	BHU/AB/42	+
16	BHU/AB/43	-
17	BHU/AB/50	-
18	BHU/AB/51	-
19	BHU/AB/52	+
20	BHU/AB/53	+
21	BHU/AB/54	-
22	BHU/AB/55	-
23	BHU/AB/56	-
24	BHU/AB/57	+
25	BHU/AB/58	-
26	BHU/AB/59	-
27	BHU/AB/62	+
28	BHU/AB/65	-
29	BHU/AB/66	+
30	BHU/AB/67	-
31	BHU/AB/68	-
32	BHU/AB/69	-
33	BHU/AB/70	-
34	BHU/AB/71	-
35	BHU/AB/72	-
36	BHU/AB/73	+
37	BHU/AB/74	-
38	BHU/AB/75	-
39	BHU/AB/76	+
40	BHU/AB/77	+

41	BHU/AB/78	+
42	BHU/AB/79	-
43	BHU/AB/80	-
